# Lactylation in Colorectal Cancer: Regulatory Networks, Functional Mechanisms, and Clinical Translational Potential

**DOI:** 10.3390/ijms27104480

**Published:** 2026-05-16

**Authors:** Diao Wei, Min Zhang, Tianyu Lei, Qinyong Hu

**Affiliations:** 1 Department of Oncology, Renmin Hospital of Wuhan University, No. 99 Zhangzhidong Road, Wuchang District, Wuhan 430060, China; sungod128@outlook.com (D.W.); 13548753578@163.com (M.Z.); 2 Renmin Hospital of Wuhan Economic and Technological Department Zone, Wuhan 430090, China; leitianyu@whu.edu.cn; 3 Wuhan University Heavy Ion Medicine Center, Renmin Hospital of Wuhan University, Wuhan Economic and Technological Department Zone, Wuhan 430090, China

**Keywords:** colorectal cancer (CRC), protein lactylation, Warburg effect, ferroptosis, gut microbiota

## Abstract

Protein lactylation, an emerging post-translational modification (PTM) driven by the metabolite lactate, has surfaced as an important regulatory layer contributing to the crosstalk between metabolic reprogramming and cellular functional plasticity in colorectal cancer (CRC). Within the unique “host–microbiota” symbiotic microenvironment of CRC, the Warburg effect—fueled jointly by oncogene activation and microbial metabolism—provides abundant substrates for lactylation. This modification is dynamically regulated by a complex enzymatic system comprising “Writers” (e.g., p300/CREB-binding protein [p300/CBP], alanyl-tRNA synthetase 1/2 [AARS1/2]) and “Erasers” (e.g., histone deacetylases [HDACs] and Sirtuins). Through intricate crosstalk with other PTMs, such as acetylation and ubiquitination, lactylation exerts critical regulatory effects on both the histone epigenetic landscape and non-histone protein functions. Functionally, lactylation not only drives malignant proliferation, invasion, and metastasis but also systematically remodels the immunosuppressive “cold” tumor microenvironment. Furthermore, it confers broad-spectrum resistance to chemotherapy, radiotherapy, targeted therapy, and immunotherapy by orchestrating a ferroptosis defense network, enhancing DNA damage repair (DDR), and activating protective autophagy. This review systematically synthesizes the regulatory networks and biological functions of lactylation in CRC, deeply elucidating the core mechanisms underlying therapy resistance. Finally, we discuss the clinical translational potential of lactylation as a novel diagnostic/prognostic biomarker and therapeutic target, aiming to provide new theoretical foundations and strategic directions for overcoming current bottlenecks in CRC clinical treatment.

## 1. Introduction: From the “Warburg Effect” to a New Era of Lactylation Modification

### 1.1. The Clinical Dilemma: Intersection of Metabolism and Resistance

Colorectal cancer (CRC) remains a global health burden with dismal five-year survival rates for advanced or recurrent cases [1,2,3,4]. A primary clinical bottleneck is the emergence of chemoresistance to standard agents, including oxaliplatin and 5-fluorouracil [5,6]. Furthermore, the prevalence of microsatellite stable (MSS) “cold tumors”—characterized by an immunosuppressive microenvironment and deficient T-cell infiltration—severely limits the efficacy of immune checkpoint inhibitors (ICIs) [7,8]. Central to these therapeutic hurdles is metabolic reprogramming, which serves as a fundamental driver of both disease progression and treatment evasion.

### 1.2. Evolution of Lactate: From Metabolic Waste to Signaling Hub

The metabolic hallmark of cancer cells is the “Warburg Effect,” where glycolysis is prioritized over oxidative phosphorylation (OXPHOS) regardless of oxygen availability, resulting in high lactate flux [9]. Once relegated to the status of a metabolic waste product, lactate is now recognized as a multifaceted “Lactormone”. According to the “Lactate Shuttle Theory,” lactate facilitates intercellular energy exchange and serves as a critical signaling mediator [10]. This paradigm shift has paved the way for identifying non-canonical functions of lactate, particularly its role in modulating the epigenetic and proteomic landscape through protein lactylation.

### 1.3. Protein Lactylation: A Strategic Nexus Between Metabolism and Epigenetics

In 2019, a seminal study characterized lysine lactylation as a novel post-translational modification (PTM), demonstrating that intracellular lactate fluctuations are directly transduced into histone modifications to regulate gene expression [11]. This discovery establishes a fundamental molecular link between cellular metabolism and the epigenetic landscape, inaugurating the field of “metabolic epigenetics.” Beyond its role in histone regulation, subsequent evidence has revealed that lactylation extensively modifies non-histone proteins, dictating cell fate by modulating protein stability, enzymatic activity, and subcellular localization [12,13]. Crucially, lactylation operates within a sophisticated regulatory network, engaging in complex “crosstalk” with other PTMs—such as acetylation, ubiquitination, and phosphorylation—to orchestrate the malignant progression of CRC [14]. Furthermore, lactylation has emerged as a crucial participant of tumor microenvironment remodeling, serving as a critical mediator of immune evasion and therapeutic resistance.

### 1.4. Overview of This Review

Given the burgeoning significance of protein lactylation in oncology, this review systematically dissects its pivotal role in CRC across four key dimensions:

(1) Upstream Regulatory Networks: Deconstructing the unique “oncogene-microbiota” dual-drive mechanism and the sophisticated enzymatic systems governing lactylation in CRC.
(2) Dual Functional Mechanisms: Detailing how histone epigenetic remodeling and non-histone functional switching drive tumor malignancy and the conversion of the immune microenvironment to a “cold” phenotype.
(3) Clinical Bottlenecks: Analyzing how lactylation orchestrates anti-ferroptosis defense networks and cellular dormancy to mediate broad-spectrum resistance across chemotherapy, targeted therapy, and immunotherapy.
(4) Translational Outlook: Outlining novel lactylation-based biomarkers and a three-dimensional therapeutic framework—comprising source blockade, process intervention, and downstream disruption—to provide a panoramic reference for overcoming CRC treatment barriers.

## 2. The Molecular Regulatory Network of Protein Lactylation

The levels and patterns of protein lactylation are not merely simple biochemical reactions determined by a single factor. Instead, they are precisely orchestrated by a multi-level and multi-dimensional complex network, spanning from the macro-microenvironment to micro-molecular interactions. This chapter will dissect this network in depth, covering the dual sources of lactylation substrates, the dynamic regulation of catalytic enzymes, and the intricate “crosstalk” with other key post-translational modifications (PTMs).

### 2.1. Sources of Lactylation Substrates: The Dual Drive of Endogenous and Exogenous Factors (Figure 1)

The occurrence of protein lactylation primarily depends on a sufficient supply of its direct precursor—lactate. In colorectal cancer (CRC), the establishment of this substrate pool exhibits a unique dual-drive characteristic, involving both “endogenous” and “exogenous” mechanisms.

#### 2.1.1. Endogenous Drivers: Synergy Between Oncogenes and Microenvironmental Adaptation

Classical oncogenic signaling pathways serve as the core engine. Central transcription factors, specifically c-Myc and hypoxia-inducible factor-1α (HIF-1α), synergistically upregulate nearly all key enzymes in the glycolytic pathway (e.g., hexokinase 2 [HK2], phosphofructokinase 1 [PFK1], enolase 1 [ENO1]) and lactate dehydrogenase A (LDHA). This enhances glycolysis and forcibly shunts the glucose metabolic flux toward lactate production [5,9]. In the context of *KRAS* mutations—the most prevalent genetic alteration in CRC—the downstream phosphoinositide 3-kinase/protein kinase B (PI3K/AKT) pathway is activated, further stabilizing HIF-1α. This coordination leads to a significant accumulation of critical lactylation modifications, such as H3K9la, through a dual mechanism of “increasing production” (promoting glycolysis) and “reducing consumption” (inhibiting histone deacetylase 1/2 [HDAC1/2]-mediated delactylation) [15].

Beyond classical pathways, aberrant expression of specific metabolic enzymes can independently drive lactate generation. For instance, aldolase B (ALDOB), a fructose-metabolizing enzyme highly expressed in CRC, has been identified as a key upstream engine that strongly promotes glycolysis and subsequent lactylation events by activating pyruvate dehydrogenase kinase 1 (PDK1) [16].

Furthermore, functional defects in certain structural proteins indirectly expand the lactate substrate pool by remodeling the tumor microenvironment (TME). A recently discovered mechanism involves the downregulation of structural maintenance of chromosomes protein 4 (SMC4), which triggers the overexpression of multiple glycolytic enzymes (e.g., HK2, phosphofructokinase, platelet (PFKP)]), leading to robust lactate accumulation [17]. Similarly, the deficiency of the tight junction protein Claudin-7 (Cldn7) activates the nuclear factor kappa B (NF-κB)/C-X-C motif chemokine ligand 1 (CXCL1) signaling axis to recruit neutrophils. These recruited neutrophils subsequently undergo drastic metabolic reprogramming, serving as an additional “production factory” for lactate within the TME [18].

Notably, dysregulated lipid metabolism also acts as an upstream driver of lactate accumulation. For example, proprotein convertase subtilisin/kexin type 9 (PCSK9), which is overexpressed in CRC, not only promotes the epithelial–mesenchymal transition (EMT) via the PI3K/AKT pathway but also has been confirmed to significantly upregulate glycolysis, thereby increasing lactate production and global protein lactylation levels [19].

Finally, mitochondrial functional impairment represents a critical “gating” mechanism for lactate accumulation. Low expression of the mitochondrial pyruvate carriers MPC1/2 effectively shuts the door on pyruvate entry into the tricarboxylic acid (TCA) cycle, forcing its massive conversion into lactate within the cytoplasm. This “mitochondrial gating” mechanism constitutes another pivotal upstream event driving lactylation [20].

#### 2.1.2. Exogenous Drivers: Metabolic Interplay Between Gut Microbiota and Stromal Cells

The unique anatomical location of the intestine endows colorectal cancer (CRC) with exogenous sources of lactate that are unavailable to other solid tumors.

Cross-kingdom regulation by gut microbiota: Gut dysbiosis is an essential hallmark of CRC development. Clinical investigations have confirmed that patients with early-stage CRC frequently exhibit microbial imbalance and impairment of the intestinal mucosal barrier (referred to as “leaky gut”), characterized by a depletion of butyrate-producing beneficial bacteria (e.g., *Lactobacillus* and *Bifidobacterium*) and a significant proliferation of lactate-producing pathogens (e.g., *Enterobacteriaceae* and *Fusobacterium nucleatum* [*F. nucleatum*]), which is accompanied by elevated circulating levels of bacterial products such as D-lactate and lipopolysaccharides (LPS) [21]. Such alterations in microbial structure directly lead to the abnormal accumulation of bacterial metabolites, including lactate, within the intestinal lumen. Intriguingly, these microorganisms can further amplify lactate production by reprogramming the metabolic machinery of host cells. For instance, *F. nucleatum* binds to host E-cadherin via its surface adhesin FadA, activating the Wnt/β-catenin pathway and upregulating c-Myc, thereby driving glycolysis and lactate generation [22]. Furthermore, it activates the host transcription factor SP1 to upregulate the expression of the long non-coding RNA (lncRNA) *ENO1-IT1*. This lncRNA acts as a “molecular guide” to recruit the histone acetyltransferase lysine acetyltransferase 7 (KAT7), systematically initiating the host’s glycolytic program at the epigenetic level [23]. Notably, the metabolic profile of the gut microbiota is multifaceted. Alongside pro-carcinogenic lactate, it encompasses metabolites with anti-tumor functions; for example, butyrate produced by beneficial bacteria is not only the preferred energy substrate for colonocytes—maintaining energy homeostasis via β-oxidation [24]—but also is a critical metabolic product generated through complex cross-feeding networks within the microbiota [25]. Consequently, a “microbial metabolic competition model” emerges within the CRC microenvironment, dictating the host’s epigenetic landscape: while pathogenic bacteria-derived lactate fuels p300-mediated lactylation to drive oncogenesis, commensal bacteria-derived butyrate acts as a potent competitive HDAC inhibitor, promoting acetylation and suppressing malignant proliferation.

Crucially, the quantitative relevance of gut-derived lactate remains unclear. The absolute contribution of exogenous bacterial lactate, relative to the massive endogenous lactate pool driven by the tumor’s Warburg effect, is unknown. Future studies utilizing in vivo isotopic tracing are required to quantify how much luminal lactate physically penetrates the tumor to directly modify its epigenetics.

Exosome-mediated remote metabolic regulation: Conversely, platelet-derived exosomes originating from the circulatory system facilitate the delivery of *LINC00183* into tumor cells, subsequently stabilizing the ENO1 protein to maintain elevated glycolytic flux and a continuous lactate supply. Mechanistically, *LINC00183* directly binds to the ENO1 protein and masks its K262 ubiquitination site, thereby sequestering it from proteasomal degradation [26]. This exosome-based intercellular communication represents a highly efficient vehicle for signal transduction within the tumor microenvironment (TME) [27].

Metabolic symbiosis with stromal cells: Furthermore, stromal cells within the TME serve as critical repositories of lactate. Cancer-associated fibroblasts (CAFs) have been shown to secrete substantial quantities of lactate through upregulated glycolysis. Once internalized by tumor cells, this lactate directly fuels downstream pro-carcinogenic events, such as the lactylation of anthrax toxin receptor 1 (ANTXR1) [28].

**Figure 1:**
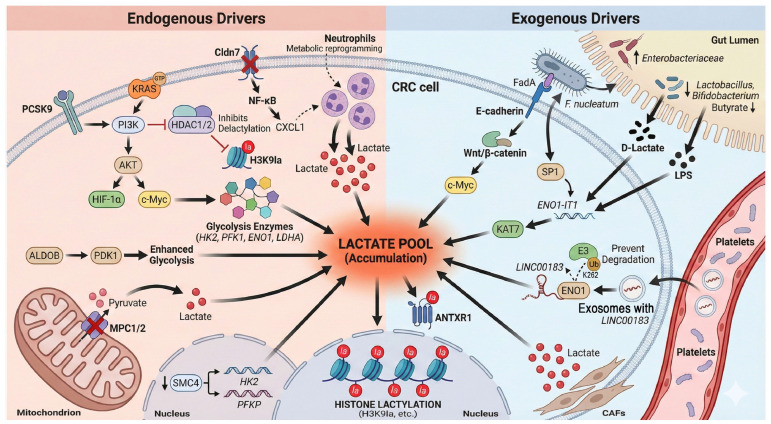
Multidimensional endogenous and exogenous mechanisms driving lactate accumulation and lactylation in colorectal cancer. This schematic illustrates the synergistic contribution of endogenous metabolic reprogramming and exogenous microenvironmental factors to the expansion of the intracellular lactate pool. Endogenously, the activation of oncogenic signaling (*KRAS*, PCSK9-PI3K/AKT) and transcription factors (c-Myc, HIF-1α) synergistically upregulates a broad spectrum of glycolytic enzymes (HK2, PFK1, ENO1, LDHA) while inhibiting HDAC-mediated delactylation. The ALDOB-PDK1 axis and mitochondrial gating defects (downregulation of MPC1/2) further force glucose flux toward lactate production. Additionally, TME remodeling triggered by the loss of structural proteins (SMC4, Cldn7) promotes glycolysis via transcriptional regulation or by recruiting metabolically active neutrophils. Exogenously, gut dysbiosis characterized by an abundance of pathogens (e.g., *F. nucleatum*, *Enterobacteriaceae*) and bacterial products (LPS, D-lactate) drives glycolysis through E-cadherin/Wnt signaling and the SP1/*ENO1-IT1*/KAT7 epigenetic axis. Platelet-derived exosomes deliver *LINC00183* to stabilize ENO1 protein by preventing its ubiquitination and degradation. Furthermore, CAFs secrete lactate directly into the TME. These converging mechanisms collectively fuel the lactate pool, serving as substrates for extensive histone (H3K9la) and non-histone lactylation (ANTXR1) to promote tumor progression. Abbreviations: CRC, colorectal cancer; PCSK9, proprotein convertase subtilisin/kexin type 9; PI3K, phosphoinositide 3-kinase; AKT, protein kinase B; HIF-1α, hypoxia-inducible factor-1α; *HK2*, hexokinase 2; *PFK1*, phosphofructokinase 1; *ENO1*, enolase 1; *LDHA*, lactate dehydrogenase A; HDAC, histone deacetylase; ALDOB, aldolase B; PDK1, pyruvate dehydrogenase kinase 1; MPC, mitochondrial pyruvate carrier; SMC4, structural maintenance of chromosomes protein 4; Cldn7, claudin-7; TME, tumor microenvironment; NF-κB, nuclear factor kappa B; CXCL1, C-X-C motif chemokine ligand 1; LPS, lipopolysaccharides; *ENO1-IT1*, ENO1-intronic transcript 1; KAT7, lysine acetyltransferase 7; CAFs, cancer-associated fibroblasts; ANTXR1, anthrax toxin receptor 1.

### 2.2. Enzymatic Regulation of Lactylation: Writers and Erasers (Figure 2)

#### 2.2.1. “Writers”

p300/CBP: The classic histone acetyltransferase p300 and its paralog, CBP (CREB-binding protein), constitute the core “writer” family responsible for catalyzing protein lactylation. A landmark study in 2019, utilizing in vitro reconstitution assays, first demonstrated that p300 utilizes lactyl-coenzyme A (Lactyl-CoA) as a substrate to transfer the lactyl group onto lysine residues of histones [11]. This discovery is rooted in the biochemical nature of p300/CBP as “promiscuous” acyltransferases; their catalytic active sites are structurally capable of accommodating and catalyzing various short-chain acyl-CoAs [14]. Due to their high degree of structural and functional redundancy, they are commonly referred to as p300/CBP. In the highly glycolytic metabolic state characteristic of CRC cells, the relative intracellular abundance of Lactyl-CoA increases, granting it a competitive advantage over acetyl-coenzyme A (Acetyl-CoA) for binding to the p300/CBP catalytic pocket, thereby leading to an upregulation of global protein lactylation levels. In specific CRC investigations, p300 has been confirmed to mediate the lactylation of several critical molecules, including PFKP [13] and histone H4K12 [5]. Notably, the catalytic activity of p300/CBP possesses significant pharmacological tractability. Specific small-molecule inhibitors, such as A-485, exert their effects by competitively binding to the catalytic site, thereby offering promising avenues for targeted therapeutic intervention [29].

Emerging “Writers”: Recent research has identified Lactyl-CoA-independent pathways for lactylation. AARS1 (alanyl-tRNA synthetase 1) directly utilizes lactate to form a high-energy lactyl-AMP intermediate, subsequently transferring the activated lactyl moiety to lysine residues on substrate proteins such as p53 [12,30]. Furthermore, lysine acetyltransferase 8 (KAT8) was recently characterized in PNAS as a novel global writer with a functional preference for lactylation over acetylation [31]. In CRC, KAT8 promotes tumor progression by lactylating substrates like eukaryotic translation elongation factor 1 alpha 2 (eEF1A2). Notably, KAT8 knockdown significantly reduces global lactylation levels and inhibits tumor growth, establishing it as a highly attractive therapeutic target [31].

#### 2.2.2. “Erasers”: The Key to Dynamic Regulation

Lactylation is a reversible process precisely governed by specific “erasers” (delactylases) that antagonize the activities of writers. Current evidence identifies the zinc-dependent histone deacetylase (HDAC) family, particularly HDAC1–3, as efficient, broad-spectrum delactylases [11]. In CRC stem cells, HDAC1 acts as the specific eraser for the critical chemoresistance marker H4K12la [6]. Interestingly, the enzymatic activity of HDAC1 is itself regulated by lactylation; modification at the K412 residue is indispensable for its canonical deacetylase function [32].

Another major class of erasers belongs to the NAD+-dependent Sirtuin family, with SIRT1-3 exhibiting robust de-lactylation activity. Within the mitochondria, SIRT3 inhibits the activity of the metabolic enzyme malic enzyme 2 (ME2) by removing its lactylation at K352, thereby disrupting tumor redox homeostasis and suppressing growth [33]. This underscores the distinct compartmentalized nature of lactylation regulation across different cellular organelles.

### 2.3. The Interplay Between Lactylation and Other Post-Translational Modifications (PTMs) (Figure 2)

Lactylation does not operate in isolation; rather, it engages in intricate “crosstalk” with classical PTMs, such as acetylation and ubiquitination, across multiple regulatory levels.

Competition for common substrates and enzymes: The most direct form of crosstalk involves competition for shared enzymatic machinery and metabolic substrates. Lactylation and acetylation share core “writers” (p300/CBP) and “erasers” (HDACs and Sirtuins), leading to direct competition at the level of their respective acyl-CoA donors (Lactyl-CoA vs. Acetyl-CoA). The hyper-glycolytic state characteristic of CRC naturally leads to the enrichment of Lactyl-CoA. This metabolic shift favors lactylation over acetylation at key histone residues, such as H3K18, thereby potentially inhibiting acetylation and reprogramming the gene transcription landscape [13,14].

Functional crosstalk with ubiquitination: Bidirectional regulation of protein stability. Lactylation modulates the ubiquitin–proteasome system (UPS) through distinct, context-dependent mechanisms. It can antagonize ubiquitination via steric hindrance at shared lysine residues, thereby safeguarding proteins from E3 ligase recognition and enhancing their stability. Conversely, lactylation may synergistically facilitate degradation by inducing conformational shifts that expose degradation motifs (degrons). This intricate interplay represents a pivotal regulatory layer for determining the stability of key effectors, such as programmed death-ligand 1 (PD-L1) and p53 [12,30].

Crosstalk with phosphorylation: Lactylation acts as an upstream signal to trigger phosphorylation cascades. Specifically, H3K18la transcriptionally upregulates aurora kinase B (AURKB), which then phosphorylates heterogeneous nuclear ribonucleoprotein M (HNRNPM), forming a “lactylation–transcription–phosphorylation” cascade [34]. Conversely, upstream pathways like PI3K/Akt directly regulate p300 activity via phosphorylation, modulating global lactylation levels.

Crosstalk with RNA modifications: Constructing multi-level epigenetic regulatory networks. Beyond protein-level PTMs, lactylation interacts with RNA epigenetic modifications—such as m6A, m5C, and m7G—to establish a molecular dialog between protein and RNA modifications. These direct molecular interactions lay the foundation for constructing downstream metabolic positive feedback loops.

Crosstalk with m6A modification: Lactylation activates the m6A system via a dual mechanism. On one hand, H3K18la transcriptionally activates the m6A “writer” methyltransferase 3 (METTL3) and “reader” YTH N6-methyladenosine RNA binding protein 2 (YTHDF2). On the other hand, lactate directly lactylates the METTL3 protein to enhance its enzymatic activity, thereby comprehensively initiating the m6A modification program [35,36].

Crosstalk with m5C and m7G modifications: Lactylation also specifically activates other RNA-modifying enzymes. For instance, H3K18la and direct protein lactylation synergistically activate the m5C “writer” NOP2/Sun RNA methyltransferase 2 (NSUN2) [37], while H3K9la activates the m7G “writer” methyltransferase 1 (METTL1) [38].

**Figure 2:**
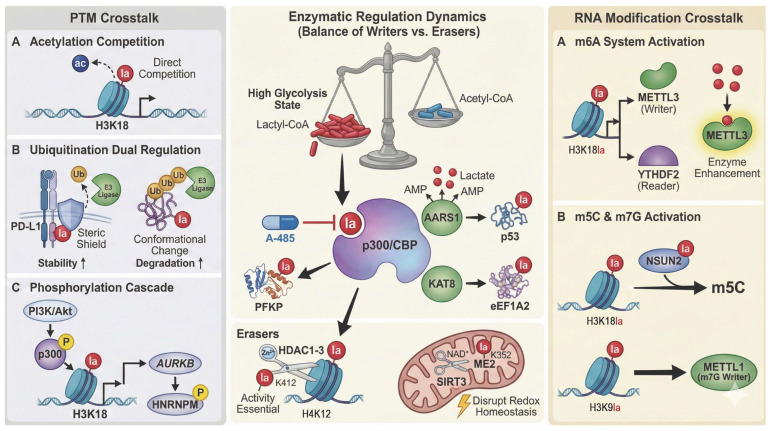
Enzymatic regulation dynamics of protein lactylation and its multidimensional crosstalk network. (Center Panel) Dynamics of Writers and Erasers: Under a highly glycolytic state, the accumulation of Lactyl-CoA competes with Acetyl-CoA, driving p300/CBP to lactylate substrates (e.g., PFKP, H4K12); this is inhibited by A-485. Novel writers include AARS1 (targeting p53) and KAT8 (targeting eEF1A2). Conversely, “Erasers” include HDAC1-3 (removing H4K12la, regulated by K412la) and mitochondrial SIRT3 (removing ME2-K352la). (Left Panel) PTM Crosstalk: Lactylation interacts with classical PTMs: (A) Acetylation Competition: Direct competition for lysine residues (e.g., H3K18). (B) Ubiquitination: Lactylation regulates stability (e.g., PD-L1) via steric shielding or conformational changes. (C) Phosphorylation Cascade: PI3K/Akt phosphorylates p300, inducing H3K18la to transcriptionally activate the AURKB-HNRNPM axis. (Right Panel) RNA Modification Crosstalk: The lactyl-epigenetic network activates RNA modifications. (A) m6A Activation: H3K18la transcriptionally upregulates METTL3/YTHDF2, while direct lactylation enhances METTL3 activity. (B) m5C and m7G: H3K18la and H3K9la cooperatively activate the writers NSUN2 (m5C) and METTL1 (m7G), respectively. Abbreviations: Lactyl-CoA, lactyl-coenzyme A; Acetyl-CoA, acetyl-coenzyme A; p300/CBP, p300/CREB-binding protein; PFKP, phosphofructokinase, platelet; AARS1, alanyl-tRNA synthetase 1; KAT8, lysine acetyltransferase 8; eEF1A2, eukaryotic translation elongation factor 1 alpha 2; HDAC, histone deacetylase; SIRT3, sirtuin 3; ME2, malic enzyme 2; PTM, post-translational modification; AURKB, aurora kinase B; HNRNPM, heterogeneous nuclear ribonucleoprotein M; m6A, N6-methyladenosine; METTL3, methyltransferase 3; *YTHDF2*, YTH N6-methyladenosine RNA binding protein 2; m5C, 5-methylcytosine; *NSUN2*, NOP2/Sun RNA methyltransferase 2; m7G, N7-methylguanosine; METTL1, methyltransferase 1.

## 3. Multidimensional Roles of Lactylation in Colorectal Cancer Progression: From Epigenetic to Protein Functional Remodeling

This regulatory framework fundamentally operates through two distinct biochemical modalities: an “epigenetic switch” via histone modifications, and a “functional switch” altering non-histone proteins. To provide a clear conceptual framework, it is essential to map these two modalities onto the key biological themes driving CRC progression. Throughout this section, we will explore how these epigenetic and functional switches synergistically orchestrate three core biological themes: (1) metabolic adaptation and autoregulatory homeostasis, (2) sustained malignant proliferation, and (3) immune microenvironment reprogramming.

### 3.1. Histone Lactylation: An “Epigenetic Switch” for Proliferation and Metabolic Adaptation

Histone lactylation acts directly on chromatin, precisely “switching” specific transcriptional programs on or off at the epigenetic level by altering chromatin structure and accessibility. This orchestrates a systematic remodeling of the malignant phenotype in CRC.

#### 3.1.1. Activating Key Oncogenes to Drive Cell Proliferation and Metabolism

H3K18la is the most extensively characterized histone lactylation mark, functioning as a potent transactivation signal at the promoters of key oncogenes in CRC. For instance, in vitro studies using human CRC cell lines (e.g., HCT116 and SW480) have demonstrated that H3K18la directly drives the transcription of *AURKB*, a key regulator of cell mitosis. The overexpression of AURKB further binds and stabilizes the RNA-binding protein HNRNPM in a kinase-independent manner, thereby preventing HNRNPM from mediating the degradation of mRNA encoding phosphoserine aminotransferase 1 (*PSAT1*), the key enzyme in the serine synthesis pathway, and ultimately providing sufficient nucleotide precursors for the rapid proliferation of tumor cells [34].

Furthermore, microbiota-derived LPS induces H3K18la to antagonize the repressor YY1, subsequently transactivating the pro-metastatic lncRNA *LINC00152* [39]. Additionally, H3K18la-mediated upregulation of insulin like growth factor 2 mRNA binding protein 2 (IGF2BP2), growth differentiation factor 15 (GDF15), and rubicon like autophagy enhancer (RUBCNL) orchestrates diverse malignant processes, including ferroptosis resistance, immunosuppression, and autophagy-mediated chemoresistance, respectively [26,40,41].

#### 3.1.2. Metabolic Adaptation: Establishing Positive Feedback Loops to Amplify the “Warburg Effect”

Histone lactylation serves as the central hub for constructing multiple “metabolic–epigenetic–metabolic” positive feedback loops. These regulatory circuits effectively “lock” tumor cells into a persistent, malignant state of high glycolysis, thereby continuously reinforcing the “Warburg Effect”.

Dual glycosylation–lactylation cycle: For instance, in the ladybird homeobox 2 (LBX2)-glutamine-fructose-6-phosphate transaminase 2 (GFPT2)-mammalian target of rapamycin complex 1 (mTORC1) axis, lactate-driven H4K12la activates the transcription factor LBX2, which subsequently upregulates GFPT2 to increase global O-GlcNAc glycosylation levels. This glycosylation modification activates mTORC1 signaling, which further promotes glycolysis and lactate production, thereby forming a malignant cycle that tightly couples these two metabolic modifications [42].

Signaling pathway–lactylation cycle: Another representative example is the golgi phosphoprotein 3 (GOLPH3)-smoothened (SMO)-AMP-activated protein kinase (AMPK) circuit. Lactate-driven H3K18la activates the transcription of *GOLPH3*, which enhances glycolysis by activating the AMPK signaling pathway. The resulting lactate, in turn, maintains H3K18la levels, leading to the continuous amplification of the metabolic signal [43].

RNA modification–lactylation cycle: As previously described, the crosstalk between lactylation and m5C (via the NSUN2 axis) or m7G (via the METTL1 axis) constitutes a potent positive feedback loop. By stabilizing the mRNA of key glycolytic enzymes (*ENO1*, *PKM2*), these circuits drive the cascade amplification of the “Warburg effect” [37,38].

Additionally, the golgi phosphoprotein 2 (GP73)-c-Myc/signal transducer and activator of transcription 3 (STAT3)-H3K18la positive feedback loop identified in hepatocellular carcinoma illustrates the interaction between lactylation and classical oncogenic signaling, a mechanism that potentially operates in CRC as well [44]. The aforementioned feedback networks effectively “lock” CRC cells into a malignant, hyper-glycolytic metabolic state, ultimately culminating in the massive accumulation of lactate within the tumor microenvironment (TME).

Notably, the core enzyme LDHA itself undergoes lysine lactylation (e.g., at K81 and K318), which directly enhances its enzymatic activity. This creates a critical positive feedback loop: initial lactate accumulation triggers LDHA lactylation, thereby further accelerating lactate production and sustainably amplifying the global lactylation landscape [45].

#### 3.1.3. Inhibiting Tumor Suppressor Genes and Releasing Growth Inhibitory Signals

The regulation of gene expression by histone lactylation is bidirectional. Beyond functioning as a transcriptional activation signal that drives oncogene expression, it can also suppress the transcription of tumor suppressor genes through epigenetic silencing. A representative example involves tumor-derived lactate inducing p300-mediated H3K18la enrichment at the promoter region of the tumor suppressor retinoic acid receptor gamma (*RARγ*), thereby repressing its transcription. The subsequent downregulation of RARγ relieves its inhibitory effect on the TNF receptor associated factor 6 (TRAF6)-interleukin 6 (IL-6)-STAT3 inflammatory axis, leading to the sustained activation of inflammatory signaling that drives the initiation and progression of CRC [46].

### 3.2. Non-Histone Lactylation: A “Molecular Functional Switch” in Tumor Progression and Survival

Beyond macro-level regulation of gene transcription, lactylation directly modifies a vast repertoire of intracellular non-histone proteins. By modulating their enzymatic activity, protein stability, or protein–protein interactions (PPIs), lactylation enables the rapid and precise orchestration of various cellular functions.

#### 3.2.1. Modulating Enzymatic Activity: Fine-Tuning Metabolic Pathways

Excessive glycolytic flux drives PFKP lactylation at K688, directly inhibiting this rate-limiting enzyme. This negative feedback loop allows tumor cells to balance high metabolic rates with environmental acid stress [13]. Conversely, mitochondrial ME2 lactylation at K352 enhances its activity and NADPH production. SIRT3-mediated de-lactylation reverses this effect, thereby disrupting redox homeostasis and suppressing tumor growth [33]. These contrasting mechanisms highlight the precise, substrate- and organelle-specific roles of lactylation in metabolic control.

#### 3.2.2. Modulating Protein Stability: The Interplay with Ubiquitination

Antagonizing ubiquitination to stabilize oncogenic proteins: A prominent example is the immune checkpoint molecule PD-L1. Research utilizing CRC cell lines and murine tumor models (such as xenografts) indicates that lactylation of the PD-L1 intracellular domain effectively prevents its ubiquitination and subsequent lysosomal degradation. This “protective” modification substantially stabilizes PD-L1 expression on the tumor cell surface, thereby intensifying inhibitory signaling to T cells—a critical step in tumor immune evasion [12].

Synergizing with ubiquitination to promote protein degradation: Lactylation can also function as a negative regulator. For the “guardian of the genome” p53, lactylation at K120/K139, catalyzed by the writer AARS1/2, disrupts its DNA-binding capacity and transcriptional activity. This conformationally aberrant p53 is preferentially recognized by the E3 ligase MDM2 proto-oncogene (MDM2)for ubiquitination-mediated degradation. Consequently, lactylation and ubiquitination synergistically ensure the silencing of the p53 tumor-suppressive pathway [12,30].

#### 3.2.3. Regulating Protein–Protein Interactions and Cellular Functions

Cytoskeletal Proteins and Signal Transduction: A quintessential example is MOESIN. In Treg cells, MOESIN lactylation at K72 significantly enhances its interaction with transforming growth factor beta (TGF-β) receptor I, thereby activating downstream SMAD family member 3 (SMAD3)signaling and reinforcing immunosuppressive functions [47].

Repair Proteins and Complex Assembly: Lactylation also facilitates the assembly of DNA damage repair (DDR) complexes. For instance, it enhances meiotic recombination 11 (MRE11) binding to DNA and promotes the recruitment of nibrin (NBS1) into the MRE11-RAD50 double strand break repair protein (RAD50)-NBS1 (MRN) complex [12]. This serves as a critical mechanism for tumor cells to counteract genomic instability and contributes to chemotherapy resistance.

Invasion and Metastasis: Lactylation drives the epithelial–mesenchymal transition (EMT). This aligns with the classical invasion–metastasis cascade theory, whereby tumor cells acquire motility and evade immune surveillance through EMT [48].

To facilitate a comprehensive understanding of this complex epigenetic and post-translational landscape, we have summarized the currently identified lactylation sites, their modifying enzymes, and their pathophysiological consequences for both histone and non-histone proteins in Table 1.

### 3.3. Remodeling the Tumor Immune Microenvironment (TIME): The Important Contributor of a “Cold” Immune Ecosystem (Figure 3)

The dual regulatory mechanisms of histone and non-histone lactylation not only autonomously drive the malignant proliferation and metastasis of CRC cells but also systematically orchestrate an immunosuppressive “cold” tumor microenvironment by modulating various immune cell subsets. Consequently, lactylation serves as a central driver of immune evasion in CRC.

#### 3.3.1. Reprogramming Myeloid Cells to Weaken Innate Immune Defenses

Myeloid cells are pivotal components of the tumor microenvironment (TME). Lactylation subverts these cells into promoters of tumor progression through multiple pathways.

Tumor-Associated Macrophages (TAMs): Tumor-derived lactate induces H3K18la modifications in macrophages, which “locks” their polarization state through a bimodal regulatory mechanism. On one hand, lactylation promotes M2 polarization via the Lactate-IGF2BP2-nuclear factor erythroid 2-related factor 2 (Nrf2) axis; on the other hand, it inhibits anti-tumor M1 polarization by directly modifying the K62 residue of pyruvate kinase M2 (PKM2). This synergy establishes a stable immunosuppressive TME [49]. Furthermore, a central “Lactate-H3K18la-RARγ” epigenetic switch represses *RARγ* expression, thereby de-repressing the TRAF6-NF-κB inflammatory pathway. This transforms macrophages into “pro-inflammatory factories” that continuously secrete tumor-promoting factors such as IL-6, driving the initiation and progression of CRC [46].

Myeloid-Derived Suppressor Cells (MDSCs): Lactylation directly promotes the recruitment of MDSCs. Through the myeloid cell nuclear differentiation antigen (MNDA)-E1A binding protein p300 (EP300)-C-X-C motif chemokine receptor 2 (CXCR2)-H3K18la axis, lactylation upregulates the chemokine receptor CXCR2 on polymorphonuclear myeloid-derived suppressor cells (PMN-MDSCs), thereby facilitating the massive infiltration of these potent immunosuppressive cells into the tumor site and establishing an inhibitory barrier [50].

Dendritic Cells (DCs): The downregulation of the mitochondrial pyruvate carrier (MPC) in tumor cells and the resulting lactate efflux drive H3K18la modification within surrounding DCs. This modification specifically activates the transcription of the inhibitory receptor *CD33*, a hallmark of DC immaturity that directly impairs antigen-presentation capacity and abrogates the initiation of anti-tumor immune responses at the source [20].

#### 3.3.2. Inhibiting Lymphocyte Function: Dismantling Adaptive Immunity

Lymphocytes are primary anti-tumor effectors, yet lactylation comprehensively dismantles their activity. High lactate levels drive MOESIN K72 lactylation in Tregs, enhancing TGF-β receptor interaction to reinforce immunosuppression [46]. Simultaneously, lactate induces PD-1 expression on Tregs via nuclear factor of activated T cell (NFAT) inhibition, further exacerbating immune evasion [51].

Moreover, abundant lactate produced by tumor cell metabolism downregulates IFN-γ expression by inhibiting the activation of NF-κB and NFAT signaling pathways, thereby suppressing the function of NK cells and T cells, inducing immune cell apoptosis, and ultimately mediating tumor immune evasion [5]. High glycolytic tumor cells also reduce the secretion of chemokines like CXCL10, restricting effector T cell infiltration and intensifying the “cold” tumor microenvironment [52].

**Figure 3:**
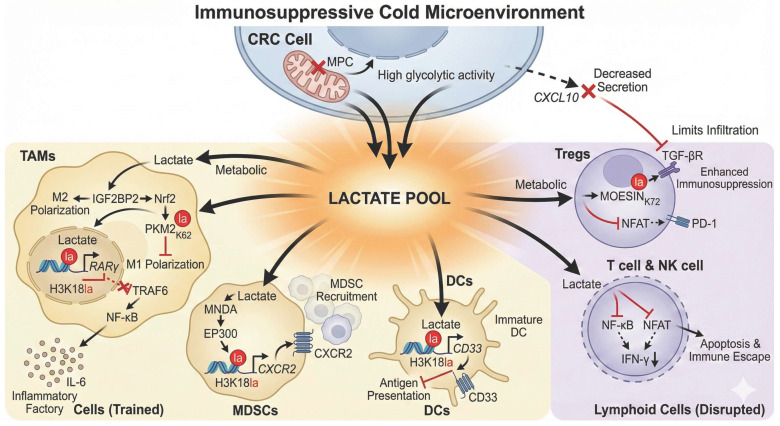
Mechanisms of colorectal cancer (CRC) reprogramming the tumor microenvironment to construct an immunosuppressive “cold” ecosystem via lactylation. This schematic illustrates how the massive Lactate Pool generated by metabolically reprogrammed CRC cells systematically orchestrates immune evasion. Metabolically, the downregulation of mitochondrial pyruvate carrier (MPC) drives lactate efflux while reducing the secretion of the chemokine CXCL10, thereby physically restricting the infiltration of effector T cells to maintain a “cold” TME. In Myeloid Cells (Left Panel), lactate subverts innate immunity: ① In TAMs, it locks the polarization state via a bimodal mechanism—promoting M2 polarization through the IGF2BP2-Nrf2 axis while inhibiting M1 polarization via PKM2-K62la. Furthermore, an epigenetic switch involving H3K18la-mediated repression of *RARγ* activates the TRAF6-NF-κB pathway, turning TAMs into IL-6-secreting inflammatory factories. ② In MDSCs, lactate activates the MNDA-EP300 axis to induce H3K18la, upregulating CXCR2 for massive recruitment. ③ In DCs, lactate-driven H3K18la specifically activates *CD33* transcription, impairing antigen presentation. In Lymphoid Cells (Right Panel), lactate dismantles adaptive immunity: ① In Tregs, it reinforces immunosuppression by lactylating MOESIN (K72) to enhance TGF-β signaling and induces PD-1 expression via NFAT inhibition. ② In Effector T and NK cells, lactate inhibits NF-κB and NFAT signaling, downregulating IFN-γ and inducing apoptosis to mediate immune escape. Abbreviations: CRC, colorectal cancer; TME, tumor microenvironment; MPC, mitochondrial pyruvate carrier; TAMs, tumor-associated macrophages; M2, M2-type macrophage; M1, M1-type macrophage; IGF2BP2, insulin like growth factor 2 mRNA binding protein 2; Nrf2, nuclear factor erythroid 2-related factor 2; PKM2, pyruvate kinase M2; *RARγ*, retinoic acid receptor gamma; TRAF6, TNF receptor associated factor 6; NF-κB, nuclear factor kappa B; IL-6, interleukin 6; MDSCs, myeloid-derived suppressor cells; MNDA, myeloid cell nuclear differentiation antigen; EP300, E1A binding protein p300; CXCR2, C-X-C motif chemokine receptor 2; DCs, dendritic cells; CD33, cluster of differentiation 33; Tregs, regulatory T cells; TGF-β, transforming growth factor beta; PD-1, programmed cell death protein 1; NFAT, nuclear factor of activated T cells; NK cells, natural killer cells; IFN-γ, interferon gamma.

## 4. Lactylation-Mediated Broad-Spectrum Therapeutic Resistance: From Conventional to Immunotherapy

As a central hub linking metabolic reprogramming with cellular functional plasticity, protein lactylation acts as a significant multifaceted contributor of treatment resistance in CRC. Through a multi-layered regulatory network, lactylation enables tumor cells to circumvent the lethal effects of chemotherapy, radiotherapy, targeted therapy, and immunotherapy, serving as a critical driver of clinical failure and recurrence.

### 4.1. Chemoresistance: Constructing a Robust Survival Stronghold

Chemotherapy regimens centered on oxaliplatin and 5-fluorouracil (5-FU) remain the cornerstone of CRC treatment, exerting cytotoxicity primarily by inducing DNA damage and oxidative stress. However, lactylation systematically abrogates these therapeutic effects through sophisticated downstream mechanisms.

#### 4.1.1. Inhibiting Ferroptosis: Establishing a Multi-Layered Antioxidant Defense Network

**Figure 4:**
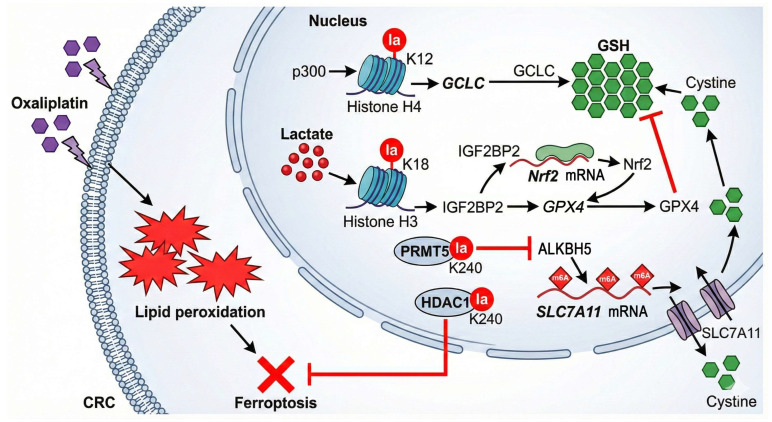
Protein lactylation orchestrates a multi-layered antioxidant defense network to inhibit oxaliplatin-induced ferroptosis in colorectal cancer cells. This schematic illustrates how lactate-driven lactylation constructs a robust defense against oxaliplatin-induced lipid peroxidation. (1) H4K12la-GCLC Axis: High lactate levels drive p300-mediated H4K12la, which transcriptionally activates *GCLC* to accelerate the synthesis of GSH (the core endogenous antioxidant). (2) H3K18la-Nrf2 Axis: H3K18la upregulates *IGF2BP2*, which binds to and stabilizes *Nrf2* mRNA. Accumulated Nrf2 protein subsequently translocates to the nucleus to activate GPX4, which utilizes GSH to scavenge toxic lipid peroxides. (3) PRMT5-SLC7A11 Axis: Lactylation of PRMT5 at K240 represses the transcription of the m6A demethylase ALKBH5. This inhibition leads to increased m6A modification and stability of *SLC7A11* mRNA, thereby enhancing cystine uptake to fuel GSH synthesis. (4) HDAC1 Axis: Lactylated HDAC1 (K240la) further fortifies this barrier by directly blocking ferroptosis execution. Collectively, these mechanisms confer chemoresistance by preventing oxidative cell death. Abbreviations: CCSCs, colorectal cancer stem cells; GCLC, glutamate-cysteine ligase catalytic subunit; GSH, glutathione; GPX4, glutathione peroxidase 4; IGF2BP2, insulin like growth factor 2 mRNA binding protein 2; Nrf2, nuclear factor erythroid 2-related factor 2; PRMT5, protein arginine methyltransferase 5; ALKBH5, alkB homolog 5; *SLC7A11*, solute carrier family 7 member 11; HDAC1, histone deacetylase 1.

Ferroptosis, an iron-dependent form of cell death driven by lipid peroxidation, is a key mechanism of oxaliplatin-induced cytotoxicity [53,54]. Lactylation establishes a robust anti-ferroptotic defense through at least four distinct signaling axes (Figure 4):

(1) The H4K12la-GCLC-GSH Axis: Preclinical evidence derived from colorectal cancer stem cells (CCSCs) and murine xenograft models reveals that high lactate levels drive p300-mediated H4K12la upregulation. This modification acts as a transcription-activating mark to directly promote the expression of glutamate-cysteine ligase catalytic subunit (GCLC), the rate-limiting enzyme for glutathione (GSH) synthesis. GSH is the most core endogenous antioxidant in cells and is essential for the catalytic function of glutathione peroxidase 4 (GPX4). Elevated GSH levels enable cells to efficiently scavenge chemotherapy-induced lipid peroxides, thus suppressing ferroptosis and conferring resistance to oxaliplatin [6].
(2) The Lactate-IGF2BP2-Nrf2-GPX4 Axis: Lactate-induced H3K18la triggers the transcription of the RNA-binding protein insulin like growth factor 2 mRNA binding protein 2 (IGF2BP2), which stabilizes nuclear factor erythroid 2-related factor 2 (*Nrf2*) mRNA to increase its protein levels. Upon nuclear translocation, Nrf2 activates various antioxidant genes, notably GPX4, directly enhancing resistance to ferroptosis [40].
(3) The PRMT5 K240lac-ALKBH5-SLC7A11 Axis: Lactylation of protein arginine methyltransferase 5 (PRMT5) at K240 represses the transcription of the N6-methyladenosine (m6A) demethylase alkB homolog 5 (ALKBH5), leading to increased m6A modification and stability of solute carrier family 7 member 11 (*SLC7A11*) mRNA. The consequent upregulation of the cystine/glutamate antiporter SLC7A11 promotes cystine uptake for GSH synthesis, thereby reinforcing the cellular anti-ferroptotic defense [55].
(4) The HDAC1 K240lac-Mediated Mechanism: Lactylation of histone deacetylase 1 (HDAC1) at the K240 residue has also been shown to confer ferroptosis resistance to CRC cells, further fortifying this defensive barrier [32].

#### 4.1.2. Enhancing DNA Damage Repair (DDR)

Chemotherapy (e.g., platinum-based agents) and radiotherapy primarily kill cancer cells by inducing DNA double-strand breaks (DSBs). Lactylation confers robust “self-repair” capabilities to tumor cells by systematically reinforcing DDR pathways.

Mechanism: Multiple key proteins within the DDR pathway are regulated by lactylation: meiotic recombination 11 (MRE11) (K673la) enhances damaged DNA binding and cleavage activity; NBS1 (K388la) facilitates the assembly of the MRN repair complex; and X-ray repair cross complementing 1 (K247la) promotes its nuclear translocation [12].

Consequences: These modifications synergistically boost the efficiency of homologous recombination (HR) and non-homologous end joining (NHEJ). This enables CRC cells to rapidly repair lethal DNA lesions induced by chemo- or radiotherapy, ultimately leading to clinical treatment failure.

#### 4.1.3. Inducing Drug Efflux and Cellular Dormancy via the SMC4/H4K12la Axis

Structural maintenance of chromosomes protein 4 downregulation serves as a critical switch to trigger glycolytic flux and lactate-mediated H4K12la elevation. This epigenetic axis promotes chemo-evasion through two sophisticated strategies.

Active Drug Efflux: It transcriptionally activates ATP-binding cassette (ABC) transporters (e.g., *ABCC2*, *ABCC3*, and *ABCC10*), which actively pump out chemotherapeutic agents such as irinotecan and oxaliplatin.

Metabolic Quiescence: It induces a transition into a “ diapause-like cancer cells (DLCCs)” dormant state. These cells, characterized by minimal proliferation and metabolic activity, effectively evade cytotoxic therapies and serve as a primary reservoir for future tumor recurrence [17].

#### 4.1.4. Promoting Tumor Stemness

Lactate derived from cancer-associated fibroblasts (CAFs) induces anthrax toxin receptor 1 (ANTXR1) lactylation at the K453 residue in CRC stem cells. This specific modification triggers the downstream Ras homolog family member C (RhoC)/Rho associated coiled-coil containing protein kinase 1 (ROCK1)/SMAD family member 5 (SMAD5) signaling cascade, which significantly bolsters stemness traits, as evidenced by the upregulation of markers such as leucine rich repeat containing G protein-coupled receptor 5 (LGR5) and CD44. This enhanced stemness ultimately mediates therapeutic resistance to oxaliplatin [28].

### 4.2. Mediating Radioresistance

Lactylation similarly plays a protective role in the context of radiotherapy. In CRC, the lactylation-driven golgi phosphoprotein 3 (GOLPH3)-SMO-AMP-activated protein kinase (AMPK) positive feedback loop serves as a pivotal mechanism for radioresistance. GOLPH3 facilitates the membrane localization of Smoothened (SMO), which activates AMPK-mediated glycolysis. This metabolic reprogramming effectively reduces radiotherapy-induced reactive oxygen species (ROS) accumulation, thereby attenuating DNA damaging effects and conferring radioresistance [43].

### 4.3. Targeted Therapy Resistance: Activating Protective Autophagy

Anti-angiogenic therapies (e.g., bevacizumab) induce hypoxia and nutrient deprivation by blocking blood supply, forcing tumor cells to adopt protective autophagy for survival. Mechanistically, lactate-induced H3K18la selectively activates the promoter of the autophagy enhancer rubicon like autophagy enhancer (*RUBCNL*) (*Pacer*). The RUBCNL protein subsequently recruits the Class III PI3K complex via beclin 1 (BECN1), facilitating autophagosome maturation. This H3K18la-RUBCNL-BECN1 axis significantly elevates autophagic flux, enabling CRC cells to maintain energy homeostasis and survive the selective pressure of targeted therapy, ultimately leading to resistance [41].

### 4.4. Immunotherapy Resistance: From “Cold” Tumors to Checkpoint Failure (Figure 5)

The majority of CRC cases, particularly microsatellite stable (MSS) subtypes, exhibit intrinsic resistance to immune checkpoint inhibitors (ICIs). This resistance originates from lactylation-mediated orchestration of a “cold” tumor microenvironment (TME), which lacks effector T cell infiltration and is saturated with immunosuppressive signals. The specific mechanisms driving this resistance are outlined below:

(1) Target Level: PD-L1 Lactylation-Mediated “Super-Stabilization”. Lactate-driven metabolic reprogramming (e.g., induced by a serine/glycine-free diet) significantly upregulates PD-L1 expression on tumor cells. Beyond transcriptional control, lactylation enhances PD-L1 stability by antagonizing ubiquitination-mediated degradation. This modification not only increases PD-L1 abundance but also extends its half-life, creating a persistent and potent immunosuppressive “brake” signal. Consequently, conventional dosages of PD-1 antibodies may be insufficient to fully abrogate the PD-1/PD-L1 interaction, ultimately leading to therapeutic failure [12,56].
(2) Effector Level: Functional Reinforcement of Immunosuppressive Cells and Competitive Sequestration. Tregs with high PD-1 expression not only cause “competitive consumption” of PD-1 antibodies (by binding the antibodies instead of effector T cells) but may also further enhance their suppressive function through PD-1 signaling. This mechanism can lead to ICI treatment promoting tumor growth, serving as a potential mechanism for hyperprogressive disease. Meanwhile, massive infiltration of polymorphonuclear myeloid-derived suppressor cells (PMN-MDSCs) releases reactive oxygen species (ROS) and arginase, which effectively inhibit the generation of CD8+ T cells. Even if anti-PD-1 antibodies activate T cells, their cytotoxicity is effectively suppressed by these MDSCs, finally leading to treatment failure [47,51].
(3) Initiation Level: Antigen Presentation “Blind Spots” via DC Paralysis. The success of ICIs is predicated on the presence of tumor-reactive T cells. However, lactylation-mediated dendritic cell (DC) dysfunction—marked by CD33 upregulation—severs the antigen presentation process. This creates an “antigenic blind spot” where, regardless of checkpoint blockade, the lack of initial T cell priming results in a terminal deficit of effector T cells within the TME. This upstream blockade at the source of the immune response represents a fundamental driver of primary resistance in CRC [20].

**Figure 5:**
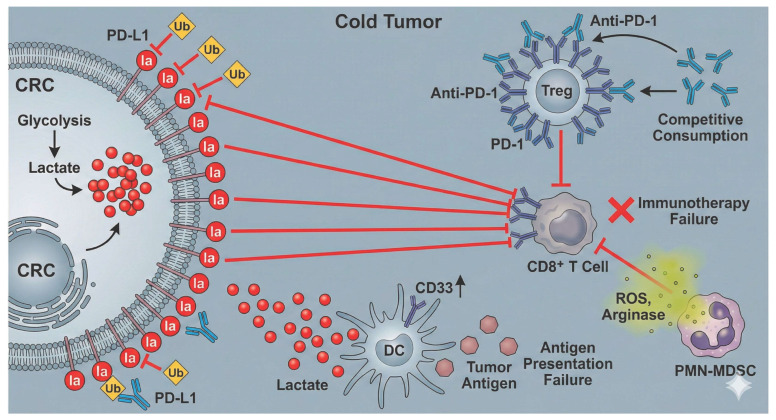
Lactylation-mediated remodeling of the tumor microenvironment drives immunotherapy resistance in colorectal cancer. This schematic illustrates how glycolysis-derived lactate and protein lactylation systematically construct an immunosuppressive “Cold Tumor” to mediate primary resistance to Anti-PD-1 therapy. Resistance mechanisms operate synergistically across three distinct levels: (1) Target Level: Lactylation of PD-L1 on CRC cells antagonizes ubiquitin (Ub)-mediated degradation, leading to PD-L1 hyper-stabilization and persistent inhibition of CD8+ T cells. (2) Initiation Level: High lactate induces CD33 upregulation on dendritic cells (DCs), creating an “antigen presentation blind spot” that severs the initial T cell priming. (3) Effector Level: Tregs expressing high PD-1 act as a “sink” for the competitive consumption of Anti-PD-1 antibodies, diverting them from effector cells; concurrently, PMN-MDSCs release ROS and arginase to directly suppress T cell cytotoxicity. Collectively, these mechanisms culminate in immunotherapy failure. Abbreviations: CRC, colorectal cancer; TME, tumor microenvironment; PD-L1, programmed death-ligand 1; PD-1, programmed cell death protein 1; Ub, ubiquitin; DCs, dendritic cells; CD33, cluster of differentiation 33; Tregs, regulatory T cells; PMN-MDSCs, polymorphonuclear myeloid-derived suppressor cells; ROS, reactive oxygen species.

## 5. Clinical Significance and Translational Potential of Lactylation (Figure 6)

Although targeting the lactylation axis offers promising therapeutic avenues for CRC, current strategies remain largely preclinical. Translating these concepts into clinical practice faces formidable biological and pharmacological barriers. This chapter delves into the potential of lactylation-associated molecules as novel biomarkers and systematically outlines multi-dimensional and multi-level therapeutic strategies centered on the lactylation regulatory network.

### 5.1. Immense Potential as Diagnostic and Prognostic Biomarkers in CRC

Given the suboptimal sensitivity and specificity of conventional biomarkers like carcinoembryonic antigen (CEA), lactylation-associated molecules—owing to their integral roles in core oncogenic processes—emerge as promising next-generation precision biomarkers for CRC.

#### 5.1.1. Tissue-Based Prognostic Biomarkers

At the tissue level, retrospective analyses of human CRC patient cohorts (e.g., using tissue microarrays) have established that specific lactylation-associated molecules serve as independent prognostic indicators for CRC. Elevated expression of ladybird homeobox 2 (*LBX2*) [42], aurora kinase B (*AURKB*) [34], insulin like growth factor 2 mRNA binding protein 2 (*IGF2BP2*) [40], and NOP2/Sun RNA methyltransferase 2 (*NSUN2*) [37] alongside deficient retinoic acid receptor gamma (*RARγ*) levels [46] are robustly linked to poor clinical outcomes. These biomarkers are mechanistically grounded: RARγ loss disinhibits inflammatory signaling, while LBX2 and AURKB drive malignant proliferation and metabolic rewiring. Furthermore, IGF2BP2 and NSUN2 facilitate progression by modulating ferroptosis and RNA modification, respectively.

Single-cell transcriptomics has further refined the identification of lactylation-rich cellular subpopulations. A notable example is the high mobility group box 2 (HMGB2)+ tumor epithelial subset, identified as a key driver of malignancy; risk models derived from this population exhibit superior prognostic accuracy [57]. Analogously, lactylation-related gene (LRG)-based molecular subtyping has proven effective in characterizing the immune heterogeneity of ulcerative colitis, thereby providing fresh insights into the immunological classification of CRC [58]. For clinical application, a 23-gene LRG score (LRGS) has been validated to accurately stratify patient survival and predict responsiveness to immunotherapy [59]. Prospectively, constructing a comprehensive expression profile comprising multiple LRGs holds promise for achieving more precise molecular stratification of CRC patients.

#### 5.1.2. Liquid Biopsy: Emerging Prospects for Non-Invasive Diagnosis and Dynamic Monitoring

Liquid biopsy offers a non-invasive solution for the early screening and longitudinal monitoring of CRC by detecting tumor-derived molecules in biofluids, such as blood and stool. Several components within the lactylation network have demonstrated significant potential as liquid biopsy biomarkers:

Exosomal lncRNAs: Plasma exosomal *LINC00183* levels correlate with advanced clinical staging and poor prognosis in CRC (AUC = 0.85). By stabilizing ENO1, it drives glycolysis and lactylation, serving as a real-time indicator of tumor metabolic activity [26]. Similarly, *LINC00152* is markedly upregulated in CRC and associated with metastasis. Given its inherent stability in biofluids, plasma *LINC00152* holds significant potential as a non-invasive biomarker for both diagnosis and prognosis (AUC = 0.82) [39].

Circulating Proteins and RNAs: Beyond exosomal markers, plasma IGF2BP2 levels have proven effective for longitudinal monitoring of therapeutic efficacy [40]. Furthermore, *tsRNA-08614* (detected in plasma or stool) and plasma *STEAP3-AS1* exhibit high predictive value for chemoresistance (AUC = 0.88) and metastatic progression (AUC = 0.85), respectively [60,61].

The discovery of these candidates paves the way for the development of non-invasive, and high-sensitivity diagnostic and surveillance toolsets for CRC.

### 5.2. Targeting the Lactylation Network: Multi-Dimensional Therapeutic Strategies

Given the central role of lactylation in CRC progression and therapeutic resistance, a quadritiered framework for intervention has emerged: upstream source blockade, intermediary process intervention, downstream effector disruption, and ecological microenvironment modulation.

#### 5.2.1. Upstream Blockade: Targeting Lactate Metabolism

This represents the most direct strategy, aiming to “starve” lactylation modifications by reducing the production or transport of lactate.

Inhibiting Lactate Production: Specific lactate dehydrogenase A (LDHA) inhibitors (e.g., FX11 [62]) or pyruvate dehydrogenase kinase (PDK)inhibitors (e.g., dichloroacetate, DCA [40]) directly suppress the conversion of pyruvate to lactate, thereby abrogating metabolic substrate availability and inducing oxidative stress [62]. While promising, the clinical translation of lactate-targeting strategies must adopt a precision medicine approach. Research in pancreatic cancer has established *TP53* mutational status as a robust biomarker for predicting FX11 efficacy [63]. Notably, LDHA inhibition exhibits high genotype dependency: *TP53*-deficient tumors are highly sensitive, whereas wild-type *TP53* maintains compensatory glycolysis via *TP53* induced glycolysis regulatory phosphatase (TIGAR) upregulation, thus attenuating the inhibitor’s effects [63]. This creates a compelling logical loop with the previously discussed mechanism where alanyl-tRNA synthetase 1 (AARS1)-mediated lactylation directly inactivates p53 [30]. High lactylation levels may mechanistically phenocopy p53 deficiency, providing a theoretical rationale for utilizing LDHA inhibitors in tumors with elevated lactylation. Consequently, clinical application necessitates a combined assessment of *TP53* mutational status and lactylation levels to precisely stratify responsive patient populations.

Blocking Lactate Transport: The application of monocarboxylate transporter 1 (MCT1)-specific inhibitors, such as AZD3965—which has progressed to Phase I clinical trials and demonstrated a favorable safety profile alongside preliminary efficacy [64]—serves to abrogate the transmembrane shuttling of lactate. This therapeutic strategy not only disrupts the metabolic symbiosis between tumor cells and stromal components, such as CAFs [28,65], but also prevents the sequestration and utilization of exogenous lactate by malignant cells, thereby exerting significant anti-tumor effects [5].

#### 5.2.2. Process-Level Intervention: Targeting Lactylation-Modifying Enzymes

Directly targeting the “writers” or “erasers” of lactylation enables more precise modulation of modification levels.

Inhibiting “Writers”: Given that p300/CBP are the primary lactylation writers, highly selective inhibitors (e.g., A-485, C646) have been shown to effectively attenuate global and site-specific (e.g., H4K12la) lactylation [29]. By reversing ferroptosis resistance in cancer stem cells, these inhibitors significantly sensitize CRC cells to chemotherapy, such as oxaliplatin [6].

Activating “Erasers”: For specific lactylation sites modulated by erasers such as sirtuin 3 (SIRT3), pharmacological activation (e.g., via Honokiol) offers a promising approach to reverse aberrant protein lactylation. For instance, Honokiol-mediated activation of SIRT3 facilitates the delactylation of the mitochondrial metabolic enzyme enzyme 2 (ME2). This modification attenuates ME2 enzymatic activity, subsequently disrupting tumor redox homeostasis and suppressing malignant growth [33].

To clearly illustrate the translational potential of targeting these enzymes, the representative pharmacological modulators and their evidenced downstream phenotypic effects in CRC are systematically organized in Table 2.

#### 5.2.3. Effector Level: Targeting Key Pathways and Combinatorial Therapies

Combination with Chemotherapy: Lactylation-mediated evasion of ferroptosis can be bypassed by combining GCLC inhibitors (e.g., buthionine sulfoximine, BSO) with oxaliplatin, which effectively reverses resistance in CRC stem cells [6]. Furthermore, co-administration of retinoic acid-inducible gene I (RIG-I) lactylation inhibitors or MCT inhibitors significantly potentiates the cytotoxic efficacy of 5-FU [5,66].

Combination with Immunotherapy: This strategy is pivotal for driving the “cold-to-hot” tumor transition. Synergizing lactylation-targeting agents (e.g., LDHA inhibitors Gossypol or FX11) with anti-PD-1 antibodies can systematically dismantle the immunosuppressive TME—for instance, by downregulating PD-1 on Tregs—thereby restoring T cell vigor in MSS CRC [12,51].

Combination with Targeted Therapy: To overcome resistance to anti-angiogenic agents like bevacizumab, the use of autophagy inhibitors (e.g., chloroquine, CQ) to block the H3K18la-RUBCNL axis-activated protective autophagy has proven effective in restoring therapeutic sensitivity [41].

#### 5.2.4. Ecological Modulation: Remodeling the Tumor Metabolic Microenvironment via Dietary Intervention and Gut Microbiota

Given CRC’s unique anatomical context, the gut microbiota and its metabolites serve as pivotal exogenous drivers of lactylation and tumor progression. Consequently, ecological modulation of the TME metabolic landscape has emerged as a highly translatable and patient-accessible therapeutic strategy.

Dietary Intervention: While serine/glycine restriction inhibits tumor growth, it triggers compensatory glycolysis and upregulates PD-L1, highlighting a synergistic potential with immunotherapy [56].

Microbiota-Based Intervention: (1) Supplementing probiotics capable of producing tumor-suppressive metabolites offers a potent strategy to reconfigure the intestinal microecology and inhibit tumor progression. For instance, supplementation with *Lactobacillus gallinarum* secretes the specific metabolite indole-3-lactic acid (ILA), which directly activates the aryl hydrocarbon receptor aryl hydrocarbon receptor (AhR) signaling pathway in CRC cells to induce apoptosis [67]. (2) Supplementing specific butyrate-producing bacteria, such as *Roseburia intestinalis*, provides the preferred metabolic substrate for intestinal epithelial cells to maintain homeostasis. Beyond providing energy, the resulting butyrate functions as an histone deacetylase (HDAC)inhibitor, which globally attenuates CRC cell lactylation while concurrently increasing acetylation levels, thereby suppressing malignant proliferation [21,68]. (3) Oral administration of manganese-engineered *Lactobacillus reuteri* can promote the accumulation of *L. reuteri* and its antitumor metabolites within the colonic tumor microenvironment [69].

Targeting Pathogenic Bacteria: The targeted elimination of lactate-producing pathogens, such as *F. nucleatum*, using antibiotics (e.g., metronidazole) can effectively disrupt the “Bacteria-*ENO1-IT1*-glycolysis” malignant axis at its origin. This intervention significantly alleviates the lactate burden and suppresses pro-tumorigenic signaling within the microenvironment [70].

Frontier Strategies: A more prospective approach involves the development of genetically engineered, “LDH-deficient” probiotics. These “designer” bacteria are programmed to consume glucose without generating lactate, thereby facilitating metabolic competition for nutrients with the tumor while concurrently attenuating lactylation levels within the microenvironment [71].

Translational Hurdles: Presenting these strategies as near-term clinical opportunities is premature. Most biomarkers lack rigorous validation in large patient cohorts. Furthermore, targeting ubiquitous enzymes (e.g., LDHA) raises severe specificity and systemic toxicity concerns. Finally, the inherent redundancy of tumor metabolism often triggers rapid compensatory pathways, limiting long-term efficacy. Addressing these hurdles is prerequisite for clinical translation.

**Figure 6:**
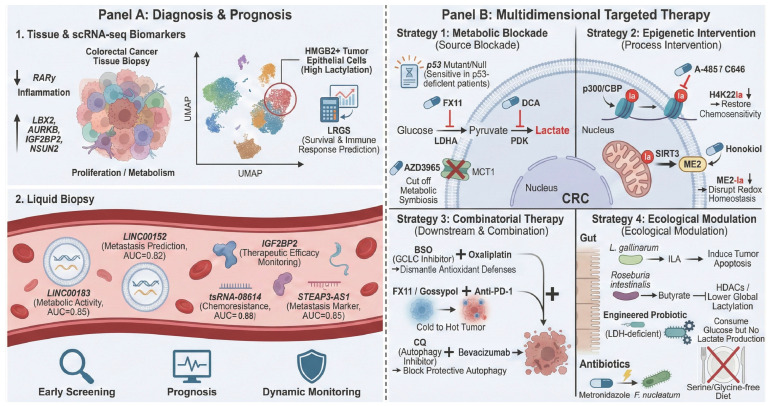
Clinical translation landscape of protein lactylation in colorectal cancer: from biomarkers to multidimensional precision therapy. This schematic illustrates the translational potential of the lactylation network. Panel (**A**): Diagnosis and Prognosis. (1) Tissue and scRNA-seq Biomarkers: Aberrant expression of specific genes (downregulated *RARγ*; upregulated *LBX2*, *AURKB*, *IGF2BP2*, *NSUN2*) and the enrichment of lactylation-high *HMGB2+* epithelial subpopulations serve as robust prognostic indicators. A lactylation-related gene score (LRGS) predicts survival and immune response. (2) Liquid Biopsy: Non-invasive biomarkers include exosomal lncRNAs (*LINC00152* for metastasis; *LINC00183* for metabolic activity), circulating proteins (*IGF2BP2* for therapeutic monitoring), and cell-free RNAs (*tsRNA-08614* for chemoresistance; *STEAP3-AS1* for metastasis), enabling early screening and dynamic surveillance. Panel (**B**): Multidimensional Targeted Therapy. (1) Metabolic Blockade: Strategies include inhibiting lactate production via FX11 (targeting LDHA, particularly effective in *TP53*-deficient tumors) or DCA (targeting PDK), and blocking transport via AZD3965 (targeting MCT1) to disrupt metabolic symbiosis. (2) Epigenetic Intervention: Targeting “writers” with A-485/C646 (p300/CBP inhibitors) decreases histone lactylation (e.g., H4K12la) to restore chemosensitivity; activating “erasers” with Honokiol (SIRT3 activator) promotes ME2 delactylation to disrupt redox homeostasis. (3) Combinatorial Therapy: BSO combined with Oxaliplatin dismantles antioxidant defenses; FX11/Gossypol combined with Anti-PD-1 reshapes the immunosuppressive microenvironment; CQ combined with Bevacizumab blocks protective autophagy to overcome resistance. (4) Ecological Modulation: Interventions include probiotics (e.g., *L. gallinarum* secreting ILA; *Roseburia intestinalis* producing butyrate), engineered LDH-deficient bacteria, antibiotics (e.g., Metronidazole targeting *F. nucleatum*), and serine/glycine-free diet to remodel the metabolic ecosystem. Abbreviations: scRNA-seq, single-cell RNA sequencing; *RARγ*, retinoic acid receptor gamma; *LBX2*, ladybird homeobox 2; *AURKB*, aurora kinase B; *IGF2BP2*, insulin like growth factor 2 mRNA binding protein 2; *NSUN2*, NOP2/Sun RNA methyltransferase 2; HMGB2, high mobility group box 2; LRGS, lactylation-related gene score; LRG, lactylation-related gene; AUC, area under the curve; *lncRNA*, long non-coding RNA; *tsRNA*, tRNA-derived small RNA; LDHA, lactate dehydrogenase A; PDK, pyruvate dehydrogenase kinase; MCT1, monocarboxylate transporter 1; SIRT3, sirtuin 3; ME2, malic enzyme 2; BSO, buthionine sulfoximine; CQ, chloroquine; ILA, indole-3-lactic acid; *AhR*, aryl hydrocarbon receptor; HDAC, histone deacetylase; *F. nucleatum*, *Fusobacterium nucleatum*; *ENO1-IT1*, ENO1-intronic transcript 1.

## 6. Conclusions and Future Perspectives

In summary, the discovery of protein lactylation has fundamentally advanced our understanding of the pathophysiological paradigm of colorectal cancer (CRC), elevating lactate from a metabolic waste product to a core signaling molecule bridging the Warburg effect and cell fate. Lactate serves as both a carbon source for tumor energetics and a precursor for histone and non-histone lactylation, driving malignant phenotypes [72]. This potency is significantly amplified within the unique CRC host–microbiota niche, where the interplay between microbial metabolites (e.g., short-chain fatty acids, SCFAs) and lactate metabolism complicates the metabolic-immune landscape [73]. Functioning as a “molecular orchestrator” through crosstalk with classical PTMs, lactylation not only fuels tumor progression but also systematically establishes a “broad-spectrum therapeutic resistance” network. For instance, METTL3 lactylation has been shown to enhance Janus kinase 1 (*JAK1*) mRNA stability to promote immunosuppressive myeloid polarization, exemplifying this novel resistance mechanism [35,74].

Despite recent progress, protein lactylation remains an expansive frontier awaiting further exploration.

### 6.1. A Unifying Conceptual Framework: The “Transduction Hub” Paradigm

**Figure 7:**
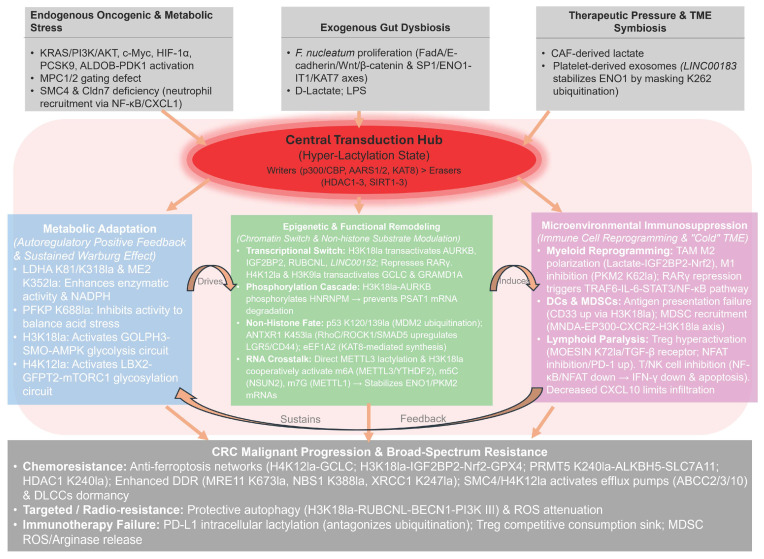
An integrative model of the lactylation-driven ecosystem in CRC. Under extreme stress, lactylation acts as a dominant central hub, synergistically orchestrating metabolic adaptation, epigenetic remodeling, and TME immunosuppression to drive CRC progression and therapeutic resistance. Abbreviations: CRC, colorectal cancer; TME, tumor microenvironment.

To provide true conceptual integration, we propose a unifying model (Figure 7). We posit that protein lactylation functions as a central transduction hub that converts extreme microenvironmental stress (e.g., therapeutic pressure or gut dysbiosis) into stable epigenetic memory. Rather than acting as isolated pathways, this lactylation-driven memory intrinsically couples metabolic adaptation, epigenetic remodeling, and microenvironmental immunosuppression into a self-sustaining, interlocking cycle. Through this paradigm, hyper-lactylation transitions from a context-specific metabolic byproduct to the dominant orchestrator of the CRC malignant ecosystem, systematically driving broad-spectrum resistance.

### 6.2. Deepening Mechanistic Research: From Histones to a Comprehensive Landscape

While the identification of lactylation “readers” is still in its infancy, non-histone lactylation—particularly on DNA repair proteins like MRE11 and XRCC1—has provided key insights into CRC chemoresistance [72]. A systematic mapping of crosstalk with other PTMs is now essential to decode the ‘modification barcodes’ underlying complex regulatory logic. Importantly, current mechanistic claims rely heavily on in vitro observations and animal models. These systems often utilize supraphysiological lactate concentrations that may not fully reflect the heterogeneous metabolic landscape of human CRC. Consequently, the reproducibility, context dependency, and generalizability of these specific signaling axes across diverse CRC molecular subtypes remain crucial questions requiring rigorous clinical validation.

To fully unlock the translational potential of lactylation, future research must address three critical knowledge gaps. First, the lack of well-defined lactylation “readers” remains a major hurdle; determining whether specific domains recognize lactylation or if it functions primarily via steric hindrance is crucial. Second, the overlap with acetylation requires deep clarification. Since both modifications compete for the same lysine residues and utilize shared enzymes (e.g., p300/CBP), decoding their dynamic competitive balance is essential. Finally, the absolute quantitative importance of lactylation in vivo remains obscure, necessitating advanced isotopic tracing in live models to definitively distinguish physiologically relevant lactylation from in vitro artifacts. Ultimately, any future clinical application of lactylation-targeted strategies must be strictly preceded by rigorous patient-based validation in large cohorts and the establishment of standardized biomarker verification protocols.

### 6.3. A Dialectical Perspective: Remodeling Immunomodulation

Lactate serves as a “double-edged sword” in immunomodulation. Beyond its inhibitory roles, lactate maintains H3K27ac via HDAC inhibition, a process essential for sustaining CD8+ T cell stemness and anti-tumor persistence [75]. Consequently, therapeutic strategies should shift from total clearance toward “metabolic homeostasis”. Examples include using lithium carbonate to restore T-cell mitochondrial function [76] or employing smart-responsive nanozymes for localized, precision lactate regulation [72].

### 6.4. Advancing Specialized Tools and Pharmacological Agents

Current therapeutic interventions are limited by the non-specificity of broad-spectrum p300 and HDAC inhibitors. To realize precision medicine, the development of highly selective small-molecule modulators targeting lactylation “writers” and “erasers” is imperative. Beyond conventional inhibition, proteolysis-targeting chimera (PROTAC) technology offers a potent strategy for the targeted degradation of key enzymes such as LDHA [72]. Furthermore, the establishment of high-fidelity antibodies and high-throughput detection platforms remains essential for translating protein lactylation into clinical biomarkers.

### 6.5. Clinical Perspectives: From Liquid Biopsy to Combination Therapy

The clinical translation of protein lactylation necessitates robust validation of its diagnostic and prognostic utility. Leveraging exosomal metabolic markers or circulating tumor DNA (ctDNA)mutations allows for real-time monitoring of CRC metabolic evolution [77], with serum lactate dehydrogenase (LDH)serving as a pivotal indicator of immunotherapy response [78]. However, a critical gap remains in the availability of longitudinal sampling datasets. Future clinical trial designs must prioritize matched pre- and post-treatment tissue or blood biopsies, coupled with concurrent spatial profiling of PD-L1 and tumor-infiltrating lymphocytes (TILs), to definitively validate the dynamic predictive value of lactylation during therapy. Crucially, therapeutic paradigms should shift toward synergistic strategies. Integrating lactylation-targeted inhibitors (e.g., LDHA/p300) with standard of care (SOC) therapies or ferroptosis inducers, especially via G protein-coupled receptor 81 (GPR81)-PD-L1 axis blockade [72], offers a potent approach to overcoming drug resistance and improving outcomes for patients with advanced CRC.

Targeting the “metabolic–epigenetic–immune“ axis represents a pivotal frontier in translational oncology, bridging the gap between fundamental biology and clinical application. This paradigm offers a strategic roadmap to dismantle the dual barriers of immune evasion and multidrug resistance (MDR) that hinder CRC therapy. Modulating this central regulatory hub holds the potential to remodel the tumor microenvironment and restore host immunity, thereby overcoming existing therapeutic plateaus and providing a new horizon for advanced CRC management.

## Figures and Tables

**Table 1:** Summary of key lactylation sites, modifying enzymes, and functional consequences in colorectal cancer.

Protein Category	Target Protein	Specific Site(s)	Validated Writer/Eraser	Core Functional Consequence	CRC-Associated Biological Effect
Histone	Histone H3	K9la	Writer: — Eraser: HDAC1/2	Transcriptional activation of target gene promoters	Drives *KRAS* mutation-mediated CRC progression via upregulating *GRAMD1A*; cooperates with *METTL1* for m7G modification
Histone	Histone H3	K18la	Writer: p300/CBP Eraser: HDAC1-3	Potent transcriptional activation mark; antagonizes transcriptional repressors (e.g., YY1)	Drives malignant proliferation (*AURKB*); promotes metastasis (*LINC00152*); mediates ferroptosis resistance (*IGF2BP2*); confers targeted therapy resistance (*RUBCNL*); remodels TME (*RARγ*)
Histone	Histone H4	K12la	Writer: p300 Eraser: HDAC1	Transcriptional activation of target genes	Promotes oxaliplatin resistance via *GCLC*; induces diapause-like cancer cell dormancy (*SMC4*); drives metabolic positive feedback loop *(LBX2*)
Non-Histone	LDHA	K81la, K318la	Writer: — Eraser: —	Enhances enzymatic catalytic activity	Establishes a positive feedback loop to sustainably amplify the Warburg effect
Non-Histone	p53	K120la, K139la	Writer: AARS1/2 Eraser: —	Inhibits DNA binding; triggers MDM2 ubiquitination	Silences the p53 tumor suppressor pathway; drives CRC tumorigenesis and chemoresistance
Non-Histone	PD-L1	Intracellular domain (lysine residues)	Writer: — Eraser: —	Antagonizes ubiquitination-mediated lysosomal degradation	Sustains high PD-L1 expression; mediates CD8+ T cell suppression and ICI resistance
Non-Histone	PFKP	K688la	Writer: p300 Eraser: —	Inhibits the enzymatic activity of this rate-limiting glycolytic enzyme	Forms a negative feedback loop to balance high glycolytic flux and environmental acid stress
Non-Histone	HDAC1	K412la, K240la	Writer: — Eraser: —	Essential for deacetylase activity (K412la); actively blocks ferroptosis execution (K240la)	K412la maintains epigenetic silencing; K240la mediates oxaliplatin resistance
Non-Histone	ME2	K352la	Writer: — Eraser: SIRT3	Enhances enzymatic activity and NADPH production	Drives CRC cell proliferation; SIRT3-mediated delactylation disrupts redox balance
Non-Histone	ANTXR1	K453la	Writer: — Eraser: —	Activates the downstream RhoC/ROCK1/SMAD5 signaling cascade	Enhances CRC stemness; mediates oxaliplatin resistance induced by CAF-derived lactate
Non-Histone	PRMT5	K240la	Writer: — Eraser: —	Represses the transcription of m6A demethylase *ALKBH5*	Increases stability of *SLC7A11* mRNA; promotes cystine uptake; confers ferroptosis resistance
Non-Histone	Moesin	K72la	Writer: — Eraser: —	Enhances interaction with TGF-β receptor I and activates SMAD3 signaling	Reinforces the immunosuppressive function of Tregs; drives CRC immune evasion
Non-Histone	eEF1A2	Multiple residues	Writer: KAT8 Eraser: —	Enhances protein synthesis efficiency	Promotes CRC cell proliferation and tumorigenesis
Non-Histone	MRE11/NBS1/XRCC1	K673la/K388la/K247la	Writer: — Eraser: —	Promotes DNA binding (MRE11), MRN assembly (NBS1), nuclear translocation (XRCC1)	Systematically reinforces DNA Damage Repair (DDR); confers resistance to chemotherapy/radiotherapy
Non-Histone	PKM2	K62la	Writer: — Eraser: —	Directly modifies and inhibits kinase activity	Inhibits anti-tumor M1 macrophage polarization; reprograms myeloid cells to construct a “cold” TME
Non-Histone	METTL3	Multiple residues	Writer: — Eraser: —	Enhances m6A methyltransferase enzymatic activity	Activates the global m6A modification program; drives immunosuppression of myeloid cells

Note: The symbol “—” indicates that the specific catalytic enzymes (writers or erasers) responsible for lactylation at these sites have not yet been conclusively identified or highlighted in the current literature. Abbreviations: *KRAS*, Kirsten rat sarcoma virus; *GRAMD1A*, GRAM domain containing 1A; *METTL1*, methyltransferase 1; *AURKB*, aurora kinase B; *LINC00152*, long intergenic non-protein coding RNA 152; *IGF2BP2*, insulin like growth factor 2 mRNA binding protein 2; *RUBCNL*, rubicon like autophagy enhancer; *RARγ*, retinoic acid receptor gamma; *GCLC*, glutamate-cysteine ligase catalytic subunit; *LBX2*, ladybird homeobox 2; LDHA, lactate dehydrogenase A; p53, tumor protein p53; PFKP, phosphofructokinase, platelet; HDAC1, histone deacetylase 1; ME2, malic enzyme 2; ANTXR1, anthrax toxin receptor 1; PRMT5, protein arginine methyltransferase 5; *ALKBH5*, alkB homolog 5; *SLC7A11*, solute carrier family 7 member 11; eEF1A2, eukaryotic translation elongation factor 1 alpha 2; MRE11, meiotic recombination 11; NBS1, nibrin; XRCC1, X-ray repair cross complementing 1; METTL3, methyltransferase 3.

**Table 2:** Pharmacological modulators targeting lactylation-modifying enzymes and their phenotypic evidence in colorectal cancer.

Enzyme Target	Pharmacological Candidate	Intervention Type	Effect on Lactylation Modification	Downstream Phenotypic Evidence in CRC
p300/CBP	A-485/C646	Inhibition	Attenuates global and site-specific (e.g., H4K12la) lactylation	Reverses ferroptosis resistance; restores chemosensitivity to oxaliplatin
SIRT3	Honokiol	Activation	Promotes delactylation of specific targets (e.g., ME2 K352la)	Disrupts tumor redox homeostasis; significantly suppresses malignant growth
HDACs	Butyrate	Inhibition	Globally attenuates CRC cell lactylation (while increasing acetylation)	Suppresses malignant cell proliferation; maintains energy homeostasis
KAT8	Specific Knockdown	Inhibition	Reduces global lactylation and specific eEF1A2 lactylation	Inhibits protein synthesis efficiency and CRC tumor growth in vivo

Abbreviations: p300/CBP, p300/CREB-binding protein; SIRT3, sirtuin 3; ME2, malic enzyme 2; HDACs, histone deacetylases; KAT8, lysine acetyltransferase 8; eEF1A2, eukaryotic translation elongation factor 1 alpha 2; CRC, colorectal cancer.

## Data Availability

No new data were created or analyzed in this study. Data sharing is not applicable to this article.
